# Supplementary material for: Invasive Phenoprofiling of Acute-Myocardial-Infarction-Related Cardiogenic Shock
Source: J Clin Med. 2023 Sep 7;12(18):5818. doi: 10.3390/jcm12185818 (PMC10532159; doi:10.3390/jcm12185818)
Supplement: Supplementary file 1 [file jcm-12-05818-s001.zip › jcm-2588952-supplementary.pdf]

Invasive Pheno-profiling of Acute Myocardial Infarction Related Cardiogenic Shock
-Supplementary data-

Supplementary Methods

Patients

We analyzed retrospective data of patients with acute myocardial infarction complicated by cardiogenic shock (AMI-CS) patients from January 2006-July 2021 at the National Institute of Cardiology in Mexico City. 309 patients with AMI-CS + PAC were included; the PAC was installed within the first 24h of CS diagnosis (median of 3 [2-5] days from the hospital admission, the cumulative percentages were 80.6%, 90.5%, and 98.9% on days 5, 7, and 10, respectively).

Classifications and definitions

According to standard definitions, patients with an acute myocardial infarction diagnosis were identified and classified as having either ST-elevation myocardial infarction (STEMI) or non-ST-elevation-myocardial infarction (NSTEMI).[1] CS was defined as a systolic pressure of ≤90 mmHg, the need for vasoactive or mechanical support (MCS), lactate ≥2 mmol/L, or a cardiac index of ≤2.2 L/min/m^2.[2]

We classified the patients according to the 2022 SCAI shock stage classification.[3] The SCAI stage was recorded as the worst at 24 h after installing a PAC monitor.

- Stage C in patients with a lactate level of at least ≥2 mmol/L at a one-time point, requiring initial intervention with either one drug or an MCS.
- Stage D was classed if the patient had a baseline lactate level >2 mmol/L plus a higher time/admission lactate >1 or deterioration in organ function, defined as an estimated glomerular filtration rate (eGFR) <45 mL/min/1.73 m^2, aspartate transaminase (AST), or alanine transaminase (ALT) levels three times the upper level (150 U/L) or a failure of initial support strategy to restore perfusion (need of an MCS plus at least one vasopressor).
- The presence of lactate ≥8 mmol/L defines stage E, pH<7.2, or an excess base <–10 mEq/L.

Hemodynamic measures

All hemodynamic recordings were completed during the installation. But as part of PAC retirement or patient death, data losses were seen at 0 h=0%, 6 h=7.4%, 12 h=12.9%, and 24 h=20.7%. The missing data were handled using the Expectation-Maximization package in IBM SPSS Statistics (v. 22; IBM Corp., Armonk, NY, USA) to input the data for the missing parameters.8

Congestion profile

We made congestion profiles in all patients using the standardized cut-off values of pulmonary capillary wedge pressure (PCWP, ≥18 mmHg) and right atrial pressure (RAP, ≥12 mmHg) to create four groups:7 biventricular, right-, left-ventricular congestion or euvolemic (EuV). As in previous studies, we used the changes in congestion profile to categorize these patients into three groups: decongestive, neutral, or congestive within the first 24 h.

Cardiometabolic phenotype

The renal best cut-off was 53.7 mL/min/1.73 m^2, Youden=0.275, a sensitivity of 62.7, and a specificity of 64.8. With a historical cut-off of 60 mL/min/1.73 m^2, the results showed a sensitivity of 70 and a specificity of 56.6%. Youden=0.27. A comparison of ROC curves and AUC showed 0.64 vs. 0.63, respectively (*P*=0.511).

Hepatic best cut-off 140 U/L, Youden=0.258, a sensitivity of 47.33, and specificity of 78.48. While a historical cut-off (>3 ULN) of 150 U/L had a sensitivity of 45.8, specificity of 79.75, and a Youden=0.26. A comparison of ROC curves with AUC showed 0.65 vs. 0.63, respectively (*P*=0.487).

Supplementary Results

Outcomes

Ventricular tachycardia or fibrillation

This outcome was defined as the presence of VT/VF that causes required cardioversion, either pharmacological or electrical.

Acute kidney injury

Acute kidney injury was classified according to the AKIN network by total Cr elevation of 0.3 or the use of hemodialysis. Basal Cr values were obtained in the electronic health record as previous Cr to the index hospitalization or the lowest Cr during the hospital stay. We estimated the Cr by a reverse eGFR to Cr formula based on the CKD-EPI equation, assuming an eGFR of 75 mL/min/1.73 m^2, as this eGFR is mentioned in the KDIGO AKI guidelines.[4]

Multi-organ failure

An in-hospital 30-day follow-up was obtained with serial measurements at days 1, 3, 5, 7, 14, and 30 days of MODS variables. The worst value was obtained for the final scoring. Where no values were available, a score of 0 was assigned to the specific organ. Since hemodynamic data were not recorded thoroughly, the worst data for the cardiovascular score were obtained using the 24-h PAC-derived measures to calculate the pressure-adjusted heart rate (PAR=HR*RAP/MAP). If the patient had sedation RASS (Richmond agitation-sedation scale), the value was converted to the Glasgow coma score (GCS).[5] Multi-organ failure was defined as two-organ involvement and a MODS value with a specific organ score of ≥2 points in each organ defined by: respiratory (PaO_2/FiO_2), renal (serum creatinine), Hepatic (bilirubin), cardiovascular (PAR), hematological (platelet count) and neurological (GCS/RASS).[6]

Hemodynamic variables and multi-organ failure, AKI, and ventricular arrhythmias (Table 3 and Supplementary Table 3S)

Multi-organ failure (Figure 1S A–C)

In the case of MOF, SBP, SBP, MAP, and perfusion pressure, there were significant differences among groups, with lower values in MOF patients. In the time-point analysis at baseline, 6, 12, and 24h, lower SBP, MAP, and perfusion pressure were seen in MOF. Only at 24h was DBP lower in this group. Regarding congestion, RAP but not PCWP had significant differences, higher in MOF; regarding RAP, there were differences only at 12 and 24h, suggesting an incomplete RV decongestion. Furthermore, for PCWP, although only a trend was seen in the ANOVA analysis, differences are present at baseline and 24h, indicating an entrance and exit with more LV congestion. As expected, lower values with statistical significance for cardiac output, index, power, power index, and $CPI_{(RAP)}$ were seen in the MOF group. SV and SVi also had similar behavior in this group, with all these hemodynamic parameters significantly different at all time points. LVSWi and RVSWi had differences more marked in LVSWi (*F*=13.55, *P*<0.001 & *F*=5.23, *P*=0.023), in the case of LVSWi. PAPi also had lower values in the MOF group in the ANOVA analysis (*F*=6.34, *P*=0.012).

AKI (Figure 1S D–F)

The SBP, MAP, and perfusion pressure were significant in the ANOVA, with lower values in patients with AKI. Regarding congestion, although no differences between groups with RAP were seen, an interaction of time*AKI was seen with a more effective decline in RAP in patients who did not have AKI. PCWP had substantial differences (F=4.42, *P*=0.036) with higher values in AKI, which was seen at 6 and 24h in the time-point analysis. Cardiac output, index and power, power index, and $CPI_{(RAP)}$ had significant differences overall lower in patients with AKI at all time points. Finally, LVSWi had differences with lower values in AKI, but in the time-point analysis, this was seen from 6 to 24h.

Ventricular tachycardia or fibrillation (Figure 1S G–I)

If ventricular tachycardia/fibrillation (VT/VF) develops, low SBP, DBP, MAP, and perfusion pressure are seen in the first 24h of active hemodynamic monitoring. In the time-point analysis, these differences were seen from 6h with DBP, MAP, and perfusion pressure; all aforementioned parameters had differences at 12-24h. Low cardiac output, index and power, power index, and $CPI_{(RAP)}$ were seen with the highest distinction made by $CPI_{(RAP)}$ (F=16.54, P<0.001). Diminished SV, SVI, and LVSWi were also seen in this VT/VF group.

References

1. Cannon, C.P.; Brindis, R.G.; Chaitman, B.R.; Cohen, D.J.; Cross, J.T.; Drozda, J.P.; Fesmire, F.M.; Fintel, D.J.; Fonarow, G.C.; Fox, K.A.; et al. 2013 ACCF/AHA Key Data Elements and Definitions for Measuring the Clinical Management and Outcomes of Patients With Acute Coronary Syndromes and Coronary Artery Disease. *J Am Coll Cardiol* **2013**, *61*, 992–1025, doi:10.1016/j.jacc.2012.10.005.

2. Jones, T.L.; Nakamura, K.; McCabe, J.M. Cardiogenic Shock: Evolving Definitions and Future Directions in Management. *Open Heart* **2019**, *6*, e000960, doi:10.1136/OPENHRT-2018-000960.

3. Naidu, S.S.; Baran, D.A.; Jentzer, J.C.; Hollenberg, S.M.; van Diepen, S.; Basir, M.B.; Grines, C.L.; Diercks, D.B.; Hall, S.; Kapur, N.K.; et al. SCAI SHOCK Stage Classification Expert Consensus Update: A Review and Incorporation of Validation Studies. *J Am Coll Cardiol* **2022**, doi:10.1016/j.jacc.2022.01.018.

4. KDIGO Clinical Practice Guideline for Acute Kidney Injury. *Kidney Int Suppl.* **2012**, *2*, 1–138.

5. Vasilevskis, E.E.; Pandharipande, P.P.; Graves, A.J.; Shintani, A.; Tsuruta, R.; Ely, E.W.; Girard, T.D. Validity of a Modified Sequential Organ Failure Assessment Score Using the Richmond Agitation-Sedation Scale. *Crit Care Med* **2016**, *44*, 138–146, doi:10.1097/CCM.0000000000001375.

6. Bota, D.P.; Melot, C.; Ferreira, F.L.; Ba, V.N.; Vincent, J.L. The Multiple Organ Dysfunction Score (MODS) versus the Sequential Organ Failure Assessment (SOFA) Score in Outcome Prediction. *Intensive Care Med* **2002**, *28*, 1619–1624, doi:10.1007/S00134-002-1491-3.

Table S1. Pairwise comparison with Bonferroni correction of the characteristics of patients with acute myocardial infarction cardiogenic shock phenotype

Variables		Cardiac-only vs. cardiorenal	Cardiac-only vs. cardiometabolic	Cardiorenal vs cardiometabolic
Age (years)		<0.001	0.001	0.353
Hypertension (%)		0.026		
Previous known CKD (%)		*	*	*
Type of AMI (%)	NSTEMI		0.022	0.035
	STEMI			
Killip-Kimball (%)	I	0.032	0.008	
	II	0.005	0.002	
	III			
	IV	<0.001	<0.001	0.024
WBC (cel/mm3)		0.046	<0.001	0.309
Neutrophils (%)		0.026	0.25	1
Lymphocytes (%)		0.046	0.103	1
Platelets (cel/mm3)		1	0.022	0.006
Glucose (mg/dL)		0.034	0.02	1
BUN (mg/dL)		<0.001	<0.001	0.562
Creatinine (mg/dL)		<0.001	<0.001	1
eGFR by CKD-EPI (ml/min/m2)		<0.001	<0.001	1
Chloride (mEq/L)		1	0.054	0.096
Potassium (mEq/L)		<0.001	<0.001	0.595
AST (U/L)		1	<0.001	<0.001
ALT (U/L)		0.196	<0.001	<0.001
LDH (U/L)		1	<0.001	<0.001
Maximum Creatinine (mg/dL)		<0.001	<0.001	0.341
Maximum AST (U/L)		1	<0.001	<0.001

Maximum ALT (U/L)		0.073	<0.001	<0.001
Bilirubin (mg/dL)		1	0.029	0.013
Minimum PaO2/FiO2 ratio		0.056	0.003	1
Lactate		0.008	<0.001	0.006
Base excess		<0.001	<0.001	0.093
pH		<0.001	<0.001	0.634
Minimum 24-h MAP		0.009	0.025	1
Minimum 24-h SBP		0.347	0.008	0.431
Primary reperfusion (<12 h, %)	Tr		0.03	
	PCI			
	NR		0.001	
Angiography (%)		0.003		
Total-PCI (%)		0.019		
Mechanical ventilation (%)			0.013	
Hemodialysis (%)		0.004	<0.001	
Vasopressin (%)			<0.001	
Dobutamine (%)		<0.001	0.011	
Levosimendan (%)			0.004	0.015
Number of vasoactive drugs (%)	None		0.016	
	1			
	2			
	3			
	4		0.001	0.014
Acute kidney injury (%)		<0.001	<0.001	
AKIN stage (%)	None	<0.001	<0.001	
	1			
	2			
	3	<0.001	<0.001	
Multi-organ failure (%)		0.004	<0.001	0.039

Number of organ failures (%)	0-1	0.021	<0.001	0.008
	2-3			
	4-5	0.004	<0.001	0.027
MODS score	0-4	0.021	<0.001	
	5-10			
	≥11	0.004	<0.001	0.027
SCAI score	C	0.011	*	*
	D			
	E	<0.001	<0.001	
Mortality (%)		0.01	<0.001	0.046

*p <0.05.

AKI: Acute Kidney Injury, ALT: Alanine Transaminase, AMI: Acute Myocardial Infarction, AST: Aspartate Transaminase, BMI: Body Mass Index, BSA: Body Surface Area, BUN: Blood Urea Nitrogen, CABG: Coronary Artery Bypass Grafting, CKD: Chronic Kidney Disease, COPD: Chronic Obstructive Pulmonary Disease, DBP: Diastolic Blood Pressure, eGFR: Estimated Glomerular Filtration Rate, HF: Heart Failure, Hs-CRP: High-sensitivity C-reactive Protein, LVEF: Left Ventricular Ejection Fraction, LDH: Lactate Dehydrogenase, MAP: Mean Arterial Pressure, MI: Myocardial Infarction, MODS: Multiple Organ Dysfunction Syndrome, NSTEMI: Non-ST-Elevation Myocardial Infarction, NR: Non-primary reperfusion, PCI: Percutaneous Coronary Intervention, PI: Pharmacoinvasive Strategy, PaO2/FiO2: Ratio of Arterial Oxygen Partial Pressure to Fraction of Inspired Oxygen, PT: Prothrombin Time, PTT: Partial Thromboplastin Time, SBP: Systolic Blood Pressure, SCAI: Society for Cardiovascular Angiography and Interventions, STEMI: ST-Elevation Myocardial Infarction, VT/VF: Ventricular Tachycardia/ Fibrillation, WBC: White Blood Cell Count

Table S2. Pairwise comparison of hemodynamics parameters in AMI-CS phenotypes

	Cardiac-only vs. cardiorenal	Cardiac-only vs. cardiometabolic	Cardioorenal vs cardiometabolic
Time 0 h			
RAP (mmHg)	0.197	0.024	1
Cardiac output (L/min)	0.2	<0.001	0.085
Cardiac index (L/min/m^2)	0.405	0.001	0.126
Cardiac power (W)	0.169	<0.001	0.117
Cardiac power index (W/m^2)	0.326	0.001	0.157
$CPI_{(RAP)}$ (W)	0.169	0.001	0.201
Perfusion pressure (mmHg)	0.1	0.087	1
Stroke volume (mL)	0.772	<0.001	0.023
Stroke volume index (mL/m^2)	1	0.002	0.05
LVSWi (gm-m/m^2/beat)	0.236	<0.001	0.111
RVSWi (gm-m/m^2/beat)	1	0.043	0.255
Time 6 h			
RAP (mmHg)	0.069	<0.001	0.312
mPAP (mmHg)	0.052	0.022	0.634
Cardiac output (L/min)	0.193	<0.001	0.011
Cardiac index (L/min/m^2)	0.406	<0.001	0.017
Cardiac power (W)	0.164	<0.001	0.05
Cardiac power index (W/m^2)	0.352	<0.001	0.059
$CPI_{(RAP)}$ (W)	0.232	<0.001	0.028
Perfusion pressure (mmHg)	0.051	0.004	1
Stroke volume (mL)	0.179	<0.001	0.059
Stroke volume index (mL/m^2)	0.401	0.001	0.077
SVR (dynes/seconds/cm^{-5})	1	0.009	0.1
PVR (dynes/seconds/cm^{-5})	0.58	0.013	0.356
SVRi (dynes/seconds*m^2/cm^{-5})	1	0.043	0.143

PVRi (dynes/seconds*m^2/cm^{-5})	0.714	0.022	0.404
LVSWi (gm-m/m^2/beat)	0.153	0.001	0.239
Time 12 h			
RAP (mmHg)	0.045	0.012	1
Cardiac output (L/min)	0.123	0.002	0.408
Cardiac index (L/min/m^2)	0.262	0.002	0.27
Cardiac power (W)	0.155	0.003	0.431
Cardiac power index (W/m^2)	0.332	0.005	0.347
$CPI_{(RAP)}$ (W)	0.22	0.001	0.239
Perfusion pressure (mmHg)	0.098	0.013	1
Stroke volume (mL)	0.203	0.003	0.361
Stroke volume index (mL/m^2)	0.414	0.006	0.308
LVSWi (gm-m/m^2/beat)	0.139	0.003	0.499
Time 24 h			
SBP (mmHg)	1	0.007	0.067
DBP (mmHg)	0.081	0.01	1
MAP (mmHg)	0.347	0.001	0.147
RAP (mmHg)	0.012	0.045	1
PCWP (mmHg)	0.02	0.003	1
PASP (mmHg)	0.054	0.135	1
PADP (mmHg)	0.153	0.001	0.263
mPAP (mmHg)	0.072	0.071	1
Cardiac output (L/min)	0.269	0.022	0.876
Cardiac index (L/min/m^2)	0.471	0.045	0.856
Cardiac power (W)	0.137	0.001	0.292
Cardiac power index (W/m^2)	0.179	0.002	0.383
$CPI_{(RAP)}$ (W)	0.053	0.001	0.497
Perfusion pressure (mmHg)	0.03	<0.001	0.48
Stroke volume (mL)	0.578	0.043	0.719

LVSWi (gm-m/m^2/beat)	0.124	0.001	0.385

Bpm: Beats per minute, SBP: Systolic blood pressure, DBP: Diastolic blood pressure, MAP: Mean arterial pressure, RAP: Right atrial pressure, PCWP: Pulmonary capillary wedge pressure, PASP: Pulmonary artery systolic pressure, PADP: Pulmonary artery diastolic pressure, mPAP: Mean pulmonary artery pressure, CPI: Cardiac power index, $CPI_{(RAP)}$: Cardiac power index normalized by right atrial pressure, PAPi: Pulmonary artery pulsatility index, SV: Stroke volume, SVi: Stroke volume index, SVR: Systemic vascular resistance, PVR: Pulmonary vascular resistance, SVRi: Systemic vascular resistance index, PVRi: Pulmonary vascular resistance index, LVSWi: Left ventricular stroke work index, and RVSWi: Right ventricular stroke work index

Table S3. ANOVA analysis of the phenotypes and complications in acute myocardial infarction complicated by cardiogenic shock.

Variables	Cardiogenic Shock Phenotypes			Multi-organ Failure			Acute Kidney Injury			Ventricular tachycardia/fibrillation		
Test	Phenotypes (F, P)	Time (F, P)	Phenotypes* time (F, P)	MOF (F, P)	Time (F, P)	MOF*time (F, P)	AKI (F, P)	Time (F, P)	AKI*time (F, P)	VT/VF (F, P)	Time (F, P)	VT/VF* time (F, P)
Heart rate (bpm)	0.44, 0.644	0.83, 0.461	0.91, 0.472	1.57, 0.212	1.4, 0.246	1.59, 0.198	0.61, 0.434	1.42, 0.241	1.47, 0.228	0.42, 0.515	0.69, 0.533	1.39, 0.248
SBP (mmHg)	3.78, 0.024	1.35, 0.259	0.24, 0.957	14.61, <0.001	1, 0.388	0.81, 0.484	4.83, 0.029	0.75, 0.514	0.59, 0.614	8.86, 0.003	1.1, 0.345	0.66, 0.571
DBP (mmHg)	2.94, 0.055	1.13, 0.337	0.35, 0.906	4.45, 0.036	0.92, 426	0.17, 0.913	3, 0.084	0.33 0.798	0.87, 0.454	8.85, 0.004	0.88, 0.446	0.2, 0.892
MAP (mmHg)	3.52, 0.031	1.36, 0.254	0.25, 0.953	11.13, 0.001	1.13, 0.334	0.29, 0.819	5.2, 0.023	0.55, 0.639	0.89, 0.439	12.39, <0.001	1.07, 0.359	0.18, 0.902
RAP (mmHg)	6.64, 0.001	0.51, 0.657	0.64, 0.683	6.26, 0.013	0.76, 0.504	0.85, 0.456	1.46, 0.227	2.03, 0.114	3.72, 0.014	0.029, 0.864	0.49, 0.675	0.21, 0.875
PCWP (mmHg)	4.72, 0.01	8.66, <0.001	0.67, 0.657	3.1, 0.079	8.32, <0.001	1.88, 0.138	4.42, 0.036	8.32, <0.001	0.93, 0.427	2.37, 0.125	10.05, <0.001	1.15, 0.324
PASP (mmHg)	2.58, 0.078	4.19, 0.006	1.8, 0.106	0.0098, 0.921	5.16, 0.002	3.25, 0.021	1.27, 0.26	6.93, <0.001	3.48, 0.02	0.7, 0.404	4.83, 0.004	0.62, 0.58
PADP (mmHg)	3.04, 0.049	3.14, 0.025	2.25, 0.037	0.4, 0.528	5.13, 0.002	2.67, 0.47	0.8, 0.372	5.85, 0.001	1.82, 0.149	1.21, 0.271	3.88, 0.012	0.61, 0.591
mPAP (mmHg)	2.79, 0.079	4.14, 0.006	0.8, 0.552	0.18, 0.671	5.83, 0.001	1.35, 0.259	2.71, 0.101	6.94, <0.001	1.93, 0.131	2.16, 0.143	5.53, 0.002	1.22, 0.3
Cardiac output (L/min)	8, <0.001	33.95, <0.001	0.99, 0.429	7.19, 0.008	32.33, <0.001	0.2, 0.878	7.69, 0.006	31.25, <0.001	0.67, 0.557	6.56, 0.011	33.28, <0.001	0.86, 0.449
Cardiac index (L/min/m^2)	5.44, 0.005	33.95, <0.001	1.01, 0.415	7.97, 0.005	32.13, <0.001	0.17, 0.897	7.14, 0.008	30.66, <0.001	0.52, 0.65	8.33, 0.004	32.81, <0.001	0.77, 0.499
Cardiac power (W)	9.92, <0.001	21.14, <0.001	0.67, 0.656	13.14, <0.001	20.8, <0.001	0.067, 0.969	12.02, 0.001	20.9, <0.001	0.53, 0.646	12.45, <0.001	20.82, <0.001	0.38, 0.747
Cardiac power index (W/m^2)	7.29, 0.001	21.39, <0.001	0.64, 0.679	14.55, <0.001	20.92, <0.001	0.047, 0.98	11.62, 0.001	20.72, <0.001	0.37, 0.755	15.25, <0.001	20.7, <0.001	0.29, 0.808
$CPI_{(RAP)}$ (W)	9.56, <0.001	18.6, <0.001	0.61, 0.708	18.57, <0.001	18.43, <0.001	0.008, 0.998	13.34, <0.001	18.94, <0.001	0.72, 0.53	16.54, <0.001	17.82, <0.001	0.16, 0.909
Perfusion pressure (mmHg)	8.17, <0.001	1.49, 0.217	0.049, 0.999	16.98, <0.001	1.42, 0.238	0.13, 0.933	6.58, 0.011	1.32, 0.268	0.59, 0.611	9.96, 0.002	1.23, 0.296	0.23, 0.859
PAPi	2.5, 0.084	0.88, 0.436	0.92, 0.467	6.34, 0.012	0.73, 0.512	0.5, 0.65	0.12, 0.727	0.56, 0.613	0.46, 0.674	0.58, 0.447	0.84, 0.456	1, 0.383
Stroke volume (mL)	6.97, 0.001	27.59, <0.001	0.97, 0.435	7.94, 0.005	25.6, <0.001	0.56, 0.613	3.78, 0.053	25.45, <0.001	0.9, 0.426	6.95, 0.009	27.59, <0.001	1.25, 0.292
Stroke volume index (mL/m^2)	4.63, 0.01	26.57, <0.001	1.01, 0.414	8.2, 0.004	24.4, <0.001	0.62, 0.578	2.82, 0.094	24.18, <0.001	0.9, 0.427	8.02, 0.005	26.35, <0.001	1.24, 0.294

SVR (dynes/seconds/cm^{-5})	3.1, 0.046	22.6, <0.001	1.45, 0.201	1.19, 0.276	20.31, <0.001	0.079, 0.962	1.66, 0.198	16.83, <0.001	0.52, 0.648	0.2, 0.655	22.85, <0.001	1.78, 0.156
PVR (dynes/seconds/cm^{-5})	2.83, 0.06	3.64, 0.016	1.08, 0.374	0.16, 0.692	4.07, 0.007	0.92, 0.421	1.88, 0.171	3.78, 0.013	1.23, 0.298	1.68, 0.196	4.71, 0.004	2.12, 0.102
SVRi (dynes/seconds*m^2/cm^{-5})	1.97, 0.142	23.77, <0.001	1.48, 0.19	1.16, 0.282	21.38, <0.001	0.098, 0.947	1.5, 0.222	17.95, <0.001	0.49, 0.665	0.75, 0.388	24.21, <0.001	1.93, 0.131
PVRi (dynes/seconds*m^2/cm^{-5})	1.86,0.157	3.58,.018	1.06, 0.382	0.14, 0.704	4.19, 0.006	1.05, 0.366	1.86, 0.174	4.02, 0.01	1.24, 0.295	1.69, 0.194	4.88, 0.004	2.24, 0.09
LVSWi (gm-m/m^2/beat)	7.18, 0.001	18.35, <0.001	0.38, 0.879	13.55, <0.001	17.84, <0.001	1.2, 0.306	7, 0.009	18.02, <0.001	0.45, 0.703	15.83, <0.001	18.26, <0.001	0.41, 0.728
RVSWi (gm-m/m^2/beat)	1.44, 0.24	3.95, 0.008	1.31, 0.257	5.23, 0.023	2.8, 0.039	1.93, 0.133	0.092, 0.761	3.06, 0.035	0.39, 0.727	0.66, 0.417	2.49, 0.068	0.013, 0.996

Bpm: Beats per minute, SBP: Systolic blood pressure, DBP: Diastolic blood pressure, MAP: Mean arterial pressure, RAP: Right atrial pressure, PCWP: Pulmonary capillary wedge pressure, PASP: Pulmonary artery systolic pressure, PADP: Pulmonary artery diastolic pressure, mPAP: Mean pulmonary artery pressure, CPI: Cardiac power index, $CPI_{(RAP)}$: Cardiac power index normalized by right atrial pressure, PAPi: Pulmonary artery pulsatility index, SV: Stroke volume, SVi: Stroke volume index, SVR: Systemic vascular resistance, PVR: Pulmonary vascular resistance, SVRi: Systemic vascular resistance index, PVRi: Pulmonary vascular resistance index, LVSWi: Left ventricular stroke work index, and RVSWi: Right ventricular stroke work index

Figure S1. ANOVA repeated measures of representative hemodynamic parameters in CS-AMI complications. In multi-organ failure, cardiac power index(RAP) (A), perfusion pressure (B), and left ventricular stroke work index (C). In acute kidney injury, cardiac power index(RAP) (D), left ventricular stroke work index (E), and perfusion pressure (F). For VT/VF (ventricular tachycardia/fibrillation), cardiac power index(RAP) (G), left ventricular stroke work index (C), and mean arterial pressure (I).

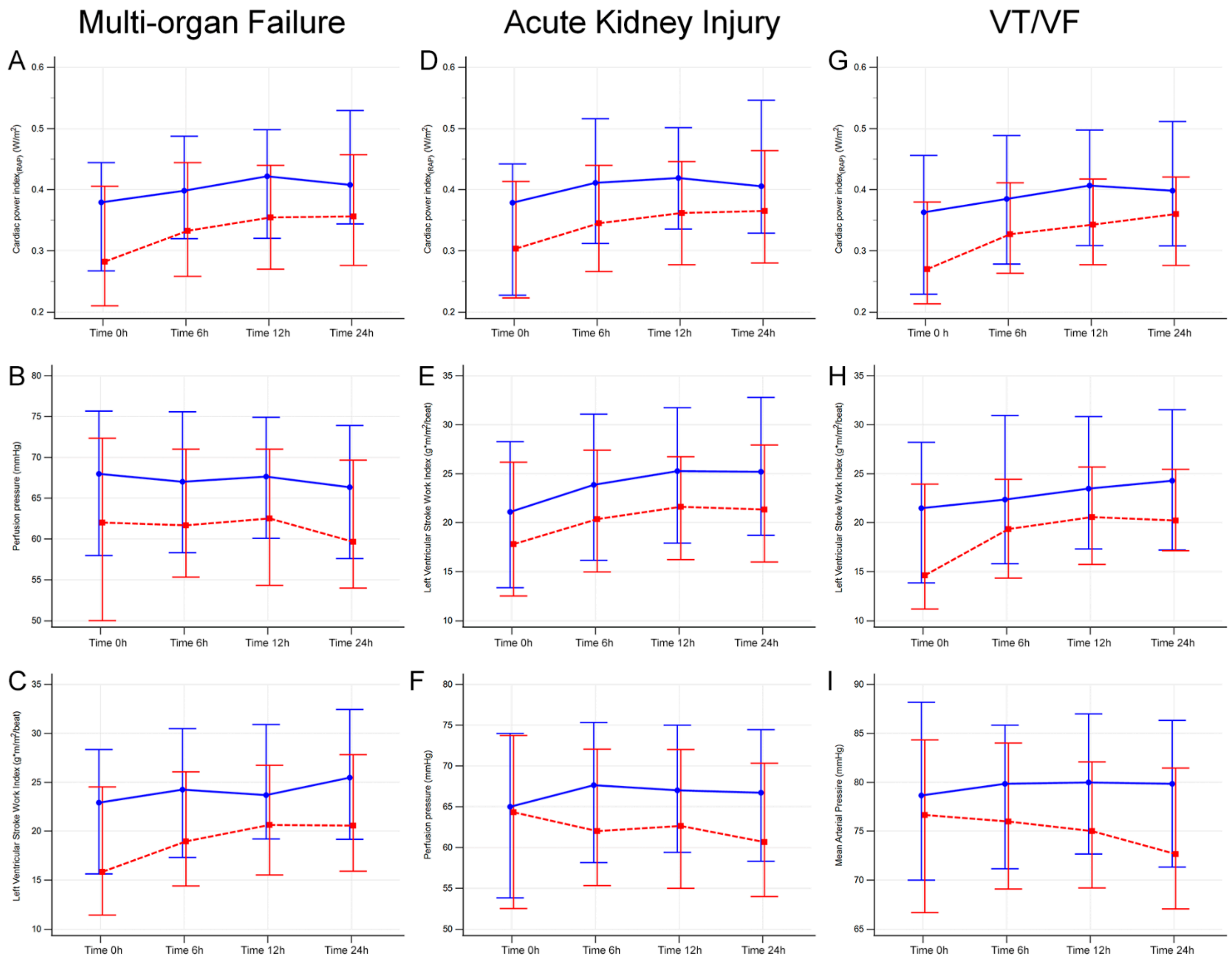

Table S4. Coronary Artery Distribution in Different Cardiogenic Shock Profiles

Variable		Cardiac only	Cardiorenal	Cardiometabolic	P-value
Left main CA		18 (14.3)	8 (10.3)	7 (10.4)	0.613
LAD		102 (81)	61 (78.2)	47 (70.1)	0.228
LCX		62 (49.2)	42 (53.8)	31 (46.3)	0.65
RCA		83 (65.9)	53 (67.9)	45 (67.2)	0.952
Number of affected vessels (%)	0	2 (1.6)	3 (3.8)	7 (10.4)	0.13
	1	41 (32.5)	24 (30.8)	18 (26.9)	
	2	43 (34.1)	21 (26.9)	21 (31.3)	
	3	40 (31.7)	30 (38.5)	21 (31.3)	

Left main: Left Main Coronary Artery, LAD: Left Anterior Descending Coronary Artery, LCX: Left Circumflex Coronary Artery, RCA: Right Coronary Artery
